# Association of HDL‐C Levels with Stroke and All‐cause Mortality: A Prospective Cohort Study from the CHARLS

**DOI:** 10.1002/brb3.71179

**Published:** 2026-03-10

**Authors:** Zhi Liu, Libo Liang, Xiao Du, He He

**Affiliations:** ^1^ Department of Laboratory Medicine, West China Hospital Sichuan University Chengdu P. R. China; ^2^ Sichuan Clinical Research Center for Laboratory Medicine Chengdu Sichuan P. R. China; ^3^ Clinical Laboratory Medicine Research Center of West China Hospital Chengdu Sichuan P. R. China; ^4^ General Practice Medical Center, West China Hospital Sichuan University Chengdu Sichuan China; ^5^ Gastric Cancer Center, West China Hospital Sichuan University Chengdu Sichuan China

**Keywords:** HDL‐C, risk, mortality, stroke

## Abstract

**Background:**

Stroke has always been a huge medical challenge faced by China. Previous studies have found a negative correlation between high‐density lipoprotein cholesterol (HDL‐C) and stroke, but in recent years, with unsatisfactory drug experiments, researchers have begun to re‐examine this relationship. Our research is based on the large population of middle‐aged and elderly people in China, exploring the relationship between HDL‐C and stroke and all‐cause mortality.

**Methods:**

The participants of this study were from the China Health and Retirement Longitudinal Study (CHARLS) database. We utilized univariate/multivariate logistic regression to screen the influencing factors of stroke. Using Cox regression, we calculated the hazard ratio (HR) for stroke and mortality, presenting our findings through Kaplan–Meier (KM) curves. We also presented the relationship between HDL‐C and stroke and mortality by restricted cubic spline (RCS) regression analysis. A time‐varying Cox analysis was conducted to evaluate the impact of changes in HDL‐C on various outcomes. To strengthen the robustness and causal interpretation, we performed subgroup analysis, sensitivity analysis, and mediation pathway, and established a competing‐risk model.

**Results:**

HDL‐C was negatively correlated with stroke. After adjusting for confounders, HDL‐C remained a protective factor for stroke (HR 0.99, 95% CI 0.99–1.00, *P *< 0.05), especially in the male group, the normal BMI group, the group without dyslipidemia, and the group with hypertension in the subgroup analysis. However, no significant association was observed between HDL‐C and all‐cause mortality after adjustment (HR 1.00, 95% CI 1.00–1.01, *p* = 0.482). The results remained robust in sensitivity analysis and competitive risk models. The impact of HDL‐C on the risk of stroke (HR 1.00, 95% CI 0.999–1.001, *p* = 0.887) and all‐cause mortality (HR 1.00, 95% CI 0.9995–1.001, *p* = 0.539) did not show a significant trend over time. Mediation analysis revealed that BMI mediated 25% of the effect of HDL‐C on stroke.

**Conclusions:**

In Chinese people over 45 years old, HDL‐C was a protective factor for stroke, while it had no significant association with all‐cause mortality. BMI mediated the effect of HDL‐C on stroke. This research highlights the critical role of HDL‐C in stroke risk assessment.

## Graphical abstract

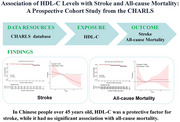

**Abbreviations**
BMI: body mass index
BUN: blood urea nitrogen
CHARLS: China Health and Retirement Longitudinal Study
CI: confidence interval
CKMS: cardiovascular‐kidney‐metabolic syndrome
Cr: creatinine
CRP: C‐reactive protein
CVD: cardiovascular disease
Cys: cystatin c
DALYs: disability‐adjusted life‐years
DBP: diastolic blood pressure
DM: diabetes mellitus
eGFR: estimated glomerular filtration rate
Glu: glucose
Hb: glycosylated hemoglobin
HDL‐C: high‐density lipoprotein cholesterol
HR: hazard ratio
ICH: intracerebral hemorrhage
IS: ischemic stroke
KM: Kaplan–Meier
LDL‐C: low‐density lipoprotein cholesterol
NHANES: National Health and Nutrition Examination Survey
RCS: restricted cubic spline
SBP: systolic blood pressure
TC: total cholesterol
TG: triglycerides
UA: uric acid

## 1 Introduction

Stroke, a well‐known cerebrovascular disease, can be classified into two main types: ischemic and hemorrhagic, with ischemic stroke (IS) accounting for up to 65.3% of all cases. From 2010 to 2021, stroke consistently ranked among the top four causes of disability‐adjusted life‐years (DALYs) and age‐standardized mortality rates globally, and it represented the greatest disease burden in East Asia (GBD 2021 Diseases and Injuries Collaborators 2024; Tsao et al. 2023). The metabolic factors linked to increased DALYs of stroke include high body mass index (BMI), elevated fasting plasma glucose, high‐sugar diets, and increased systolic blood pressure (SBP). The management of high‐risk factors and lifestyle changes can significantly reduce the risk of death caused by stroke (Johnson et al. 2016). Since 2015, the prevalence of metabolic risk factors has led to a rise in the incidence, mortality, and DALYs associated with stroke in various regions, particularly in East Asia. This trend has contributed to a growing economic burden. Not only medical expenses, but also indirect expenses of income loss are the most important burden in the cost of stroke (Demaerschalk et al. 2010). The incidence of stroke increases with age; however, there has been a concerning increase in the stroke affecting younger individuals (van Asch et al. 2010). As a result, it is critical to focus on preventing and monitoring the occurrence and progression of stroke.

HDL‐C plays a crucial role in absorbing and transporting cholesterol from peripheral tissues to the liver for metabolism. Often referred to as “good cholesterol,” HDL‐C exhibits beneficial properties such as resisting arteriosclerosis, anti‐inflammatory, antioxidant, anti‐thrombotic, and endothelial protection functions. Previous studies have indicated a negative correlation between HDL‐C levels and the risk of cardiovascular disease (including stroke) (Zhang et al. 2012; Gu et al. 2019; Sun et al. 2019). However, multiple clinical trials have shown that increasing HDL‐C levels does not effectively improve cardiovascular outcomes and may even raise the risk of adverse events, suggesting that HDL‐C may not be a reliable target for drug therapy (März et al. 2017). In recent years, some studies indicate that high levels of HDL‐C are linked to an increased risk of cardiovascular disease (CVD) and all‐cause mortality (Liu et al. 2022; Li et al. 2022).

Exploring the relationship between HDL‐C and stroke and all‐cause mortality in the middle‐aged and elderly population in China can enhance our ability to cope with stroke and negative health outcomes, which is also the main purpose of our study.

## 2 Material and Methods

### 2.1 Data Source and Study Design

**FIGURE 1:**
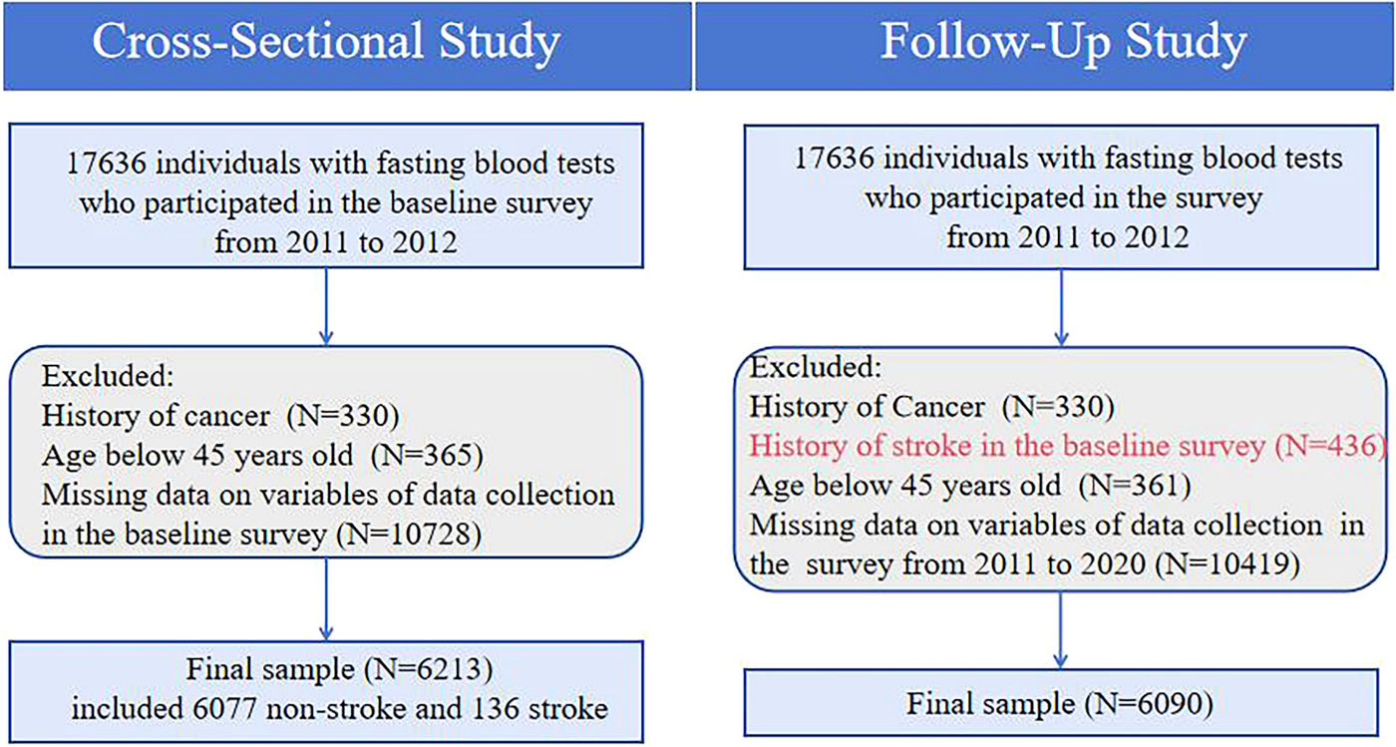
Flowchart of the study population.

We used data from the CHARLS study, a high‐quality database for the Chinese middle‐aged and elderly. CHARLS represents a nationwide investigation conducted by randomly selecting individuals aged 45 and above from households, focusing on their socioeconomic status as well as personal health conditions. The baseline survey was carried out during 2011–2012, and subsequently, follow‐up surveys were implemented in 2013, 2015, 2018, and 2020. Additionally, two blood tests were administered in 2011 and 2015, respectively (Zhao et al. 2014). Based on the characteristics of the CHARLS data, we employed both cross‐sectional analysis and longitudinal follow‐up to examine the association between HDL‐C levels and stroke risk as well as all‐cause mortality. We addressed missing data by uniformly excluding cases with missing values in the included indicators. This resulted in the inclusion of 6213 participants in the cross‐sectional analysis and 6090 participants in the follow‐up analysis. The specific sample inclusion process is shown in Figure 1.

All participants in the CHARLS study signed written informed consent, approved by the Biomedical Ethics Review Committee of Peking University (IRB00001052‐11015).

### 2.2 Assessment of Outcomes

The primary outcome of this study was the occurrence of stroke. Participants who responded “yes” to the standardized question “Have you been diagnosed with a stroke by a doctor?” were defined as individuals with stroke. Death was the second outcome variable we were concerned about.

### 2.3 Data Collection

The other disease assessments in the CHARLS research are mainly based on the respondents' self‐reports or clinical indicators for diagnosis. Hypertension is defined as SBP ≥ 140 mmHg and/or diastolic blood pressure (DBP) ≥ 90 mmHg, or self‐reported history of hypertension; diabetes mellitus (DM) is defined as fasting glucose (Glu) ≥ 126 mg/dL, or glycosylated hemoglobin (Hb) ≥ 6.5%, or self‐reported history of DM; dyslipidemia is defined as triglycerides (TG) ≥ 200 mg/dL, total cholesterol (TC) ≥ 240 mg/dL, HDL‐C < 40 mg/dL, low‐density lipoprotein cholesterol (LDL‐C) ≥ 160 mg/dL, or self‐reported history of dyslipidemia (Lin et al. 2018). The collection of medical histories for cancer, liver disease, kidney disease, digestive disease, and heart problems relies on self‐reported diagnoses by patients based on their doctor's assessments.

We not only included general clinical information such as age, gender, height, weight, waist circumference, education level, smoking history, alcohol consumption history of the subjects, and history of lipid‐lowering therapy, but also recorded their SBP, DBP, and laboratory test results, including blood urea nitrogen (BUN), uric acid (UA), creatinine (Cr), cystatin c (Cys), TG, TC, HDL‐C, LDL‐C, Glu, Hb, and C‐reactive protein (CRP).

### 2.4 Statistical Analysis

The median [interquartile range] described the distribution of non‐normally distributed continuous variables, while the frequency (percentage) described categorical variables. The Wilcoxon rank sum test or chi‐square test was used to evaluate differences between continuous or categorical variables across different groups, respectively.

In the cross‐sectional study, we assessed the association between HDL‐C and stroke by modeling it as both a continuous variable and a categorical variable based on quartiles, using univariate and multivariate logistic regression. Model 1: unadjusted; Model 2: adjusted for age, gender, smoking, alcohol consumption, and BMI; Model 3: further adjusted for age, gender, smoking, alcohol consumption, BMI, CRP, eGFR, and lipid‐lowering therapy. Covariates for Model 2 were selected based on conventional risk factors for stroke. Further covariates for Model 3 were suggested by experts and were also significant in the univariate logistic regression as factors influencing stroke. To maximize discriminative power for the outcomes of interest, the continuous HDL‐C variable was categorized for survival analysis using proper cut‐off values. These thresholds were determined with the R *surv_cutpoint* function. KM curves were used to compare survival differences between different groups, and Cox regression was used to evaluate the risk factors associated with dependent variables through HR values. To better reflect the dose‐response relationship between the independent and dependent variables, we introduced the RCS. In the RCS analysis, we used the default knot placement provided by the *rms* package in R. Higher values of both R^2^ and Dxy indicate better model fit. We compared models with 3, 4, and 5 knots and selected the optimal number based on model fit statistics. HDL‐C was measured at limited time points, introducing the potential for regression‐dilution bias. To evaluate the effect of variation in HDL‐C on the results, we conducted a time‐varying Cox analysis and assessed the correlation between HDL‐C levels measured in 2011 and 2015. For the applicability of the relationship between HDL‐C and stroke and mortality, we also conducted a subgroup analysis. To strengthen the robustness and causal interpretation, we performed sensitivity analysis, mediation pathway, and established a competing‐risk model.

All statistical analyses were performed using R software (version 4.4.1). A two‐tailed *P* < 0.05 was considered statistically significant.

## 3 Results

### 3.1 Basic Characteristics of the Study Population

In the cross‐sectional study, Table 1 presents baseline data from 2011 to 2012, including 6077 non‐stroke individuals and 136 stroke individuals. Based on the statistically significant difference (*p* < 0.05), individuals of the stroke group in the baseline data were older than the non‐stroke group, showing elevated UA, Cr, Cys, TG, Glu, and CRP, and reduced eGFR and HDL‐C, and a higher incidence of hypertension, DM, dyslipidemia, and heart problems.

**TABLE 1:** Characteristics of participants in the baseline survey from 2011 to 2012.

	Non‐Stroke	Stroke	*p*
N	6077	136	
UA (mg/dL)	4.31 [3.57, 5.18]	4.86 [4.03, 5.78]	< 0.001
Cr (mg/dL)	0.76 [0.66, 0.88]	0.84 [0.69, 0.99]	< 0.001
Cys (mg/L)	0.99 [0.86, 1.14]	1.09 [0.96, 1.29]	< 0.001
TG (mg/dL)	103.54 [74.34, 150.45]	112.39 [81.20, 173.68]	0.036
HDL‐C (mg/dL)	49.87 [40.98, 60.31]	44.46 [36.34, 55.77]	< 0.001
Glu (mg/dL)	102.06 [94.32, 112.32]	103.77 [96.62, 116.32]	0.046
CRP (mg/dL)	1.04 [0.55, 2.17]	1.48 [0.74, 3.71]	< 0.001
SBP (mmHg)	128.00 [115.33, 143.67]	133.83 [118.08, 148.33]	0.017
Weight (kg)	57.40 [50.40, 65.50]	60.60 [52.27, 69.60]	0.001
Waist (cm)	84.80 [78.00, 92.00]	90.00 [82.00, 97.60]	< 0.001
BMI (kg/m^2^)	23.05 [20.71, 25.66]	24.59 [21.81, 27.18]	< 0.001
Age (years)	60.00 [53.00, 68.00]	65.00 [58.00, 72.00]	< 0.001
eGFR (mL/min/1.73m^2^)	93.32 [82.56, 101.41]	85.10 [75.19, 94.72]	< 0.001
Smoking (%)			< 0.001
Never	3663 (60.3)	73 (53.7)	
Quit	547 (9.0)	28 (20.6)	
Current	1867 (30.7)	35 (25.7)	
Lipid‐lowering therapy (%)			< 0.001
None	5772 (95.0)	112 (82.4)	
Chinese medicine	61 (1.0)	5 (3.7)	
Western medicine	228 (3.8)	16 (11.8)	
Other treatments	16 (0.3)	3 (2.2)	
Hypertension (%)	2531 (41.6)	96 (70.6)	< 0.001
DM (%)	967 (15.9)	33 (24.3)	0.012
Dyslipidemia (%)	2527 (41.6)	80 (58.8)	< 0.001
Digestive disease (%)	1455 (23.9)	22 (16.2)	0.045
Heart problems (%)	733 (12.1)	31 (22.8)	< 0.001

Abbreviations: BMI: body mass index; Cr: creatinine; CRP: C‐reactive protein; Cys: cystatin c; DM: Diabetes mellitus; eGFR: estimated glomerular filtration rate; Glu: glucose; HDL‐C: high‐density lipoprotein cholesterol; SBP: systolic blood pressure; TG: triglycerides; UA: uric acid.

In this 9‐year follow‐up study of 6090 individuals without stroke, 528 experienced a stroke, and 816 died. Table S1 in the additional files summarizes the differences in basic data between stroke and non‐stroke individuals, as well as between the surviving and deceased populations. The study found that stroke participants were generally older with higher body weight, waist circumference, and BMI, while having lower HDL‐C levels. Additionally, they also had a higher incidence of diseases, as mentioned in Table S1. In contrast, those who died were older, predominantly male, and smokers, and had lower education levels. Interestingly, despite having lower weight, waist circumference, and BMI, they showed increased HDL‐C levels, lower TG and LDL‐C levels, and increased incidence of hypertension compared to survivors.

### 3.2 Relationship Between Baseline HDL‐C Levels and Stroke

In the baseline survey, we screened differential variables using univariate logistic regression as shown in Table 2. Analysis showed that HDL‐C and eGFR were both protective factors for stroke. We treated HDL‐C as a continuous variable and a categorical variable (divided by quartiles) and then adjusted for confounding factors. As a continuous variable, after adjusting for Model 3, HDL‐C remained a protective factor for stroke. When HDL‐C was used as a categorical variable, the risk of stroke exhibited a decreasing trend with increasing HDL‐C levels in various models, as shown in Table 3.

**TABLE 2:** Univariate logistic regression results of the baseline survey.

Index	OR	CI	*P*
UA	1.32	1.17–1.48	< 0.001
Cr	1.78	1.16–2.72	0.01
Cys	2.21	1.54–3.16	< 0.001
TG	1	0.99–1.00	0.04
HDL‐C	0.97	0.96–0.99	< 0.001
CRP	1.05	1.02–1.09	< 0.001
SBP	1.01	1.00–1.02	0.02
Weight	1.02	1.01–1.04	< 0.001
Waist	1.04	1.03–1.06	< 0.001
BMI	1.07	1.03–1.11	<0.001
Age	1.04	1.02–1.06	< 0.001
eGFR	0.97	0.96–0.98	< 0.001
Quite smoke^a^	2.57	1.65–4.01	< 0.001
Chinese medicine for dyslipidemia^b^	4.22	1.67–10.71	< 0.001
Western medicine for dyslipidemia^b^	3.62	2.11–6.21	< 0.001
Other treatments for dyslipidemia^b^	9.66	2.78–33.63	< 0.001

Abbreviations: BMI: body mass index; Cr: creatinine; CRP: C‐reactive Protein; Cys: cystatin c; eGFR: estimated glomerular filtration rate; HDL‐C: high‐density lipoprotein cholesterol; SBP: systolic blood pressure; TG: triglycerides; UA: uric acid.

^a^ Never smoking.

^b^ None lipid‐lowering therapy.

**TABLE 3:** The OR (95% CI) of stroke according to HDL‐C in three models.

Categories	Model 1	Model 2	Model 3
	OR (95%CI) *p*‐value		
Continuous HDL‐C per unit	0.97 (0.96–0.99) < 0.001	0.98 (0.97–0.99) 0.002	0.98 (0.97–1.00) 0.023
Quartile			
Q1	Ref.	Ref.	Ref.
Q2	0.63 (0.40–0.96) 0.034	0.67 (0.43–1.03) 0.072	0.75 (0.47–1.16) 0.195
Q3	0.54 (0.34–0.84) 0.008	0.61 (0.38–0.96) 0.034	0.71 (0.44–1.13) 0.159
Q4	0.34 (0.19–0.56) < 0.001	0.40 (0.22–0.68) 0.001	0.47 (0.26–0.81) 0.008

*Note*: Model 1: unadjusted; Model 2: adjusted for age, gender, smoking, drinking, and BMI; Model 3: Model 2 + adjusted for eGFR, CRP, lipid‐lowering therapy.

### 3.3 Relationships Between HDL‐C Levels and the Incidence of Stroke and Mortality During Follow‐Up

**FIGURE 2:**
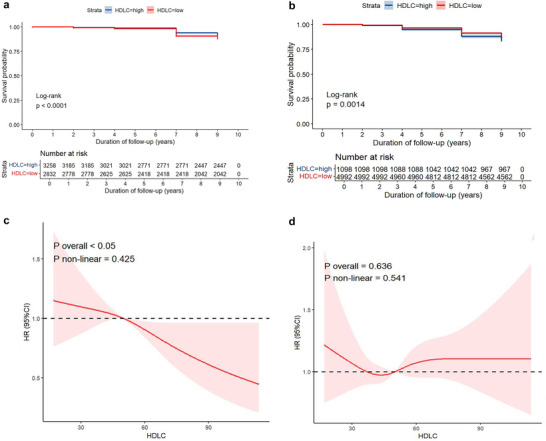
Associations between HDL‐C and stroke and mortality. (A,B) K–M curves of HDL‐C and Stroke/Mortality. (C,D) RCSs of relationships between HDL‐C and stroke/mortality.

In the follow‐up study on stroke outcomes, we established a cut‐off point of 48.33 for HDL‐C. Values above this threshold indicated high HDL‐C, while those below indicated low HDL‐C. The KM curve shown in Figure 2 demonstrated that higher HDL‐C levels were associated with a reduced likelihood of progressing to stroke, and this difference was statistically significant. The Cox regression analysis in Table S2 revealed an HR for HDL‐C of 0.98, with a 95% CI of 0.98 to 0.99 when unadjusted. After adjustment in Models 2 and 3, the HR became 0.99. Simultaneously, we set a cut‐off value of 64.18 when analyzing mortality as an outcome. Interestingly, high levels of HDL‐C became a risk factor for death (illustrated in Figure 2), yielding an HR of 1.01 (95% CI 1.00 to 1.01, *p* < 0.05) when unadjusted in Table S3. However, after adjusting in Models 2 and 3, the HR became 1.00 with no statistically significant difference.

Further investigation using RCS revealed distinct associations between HDL‐C and the two outcomes. After adjusting for covariates in Model 3, HDL‐C was independently associated with stroke risk (overall P < 0.05, nonlinear P = 0.425, modeled with 3 knots), consistent with a linear relationship. In contrast, HDL‐C showed no significant association with all‐cause mortality (overall P = 0.636, nonlinear P = 0.541, modeled with 4 knots).

**FIGURE 3:**
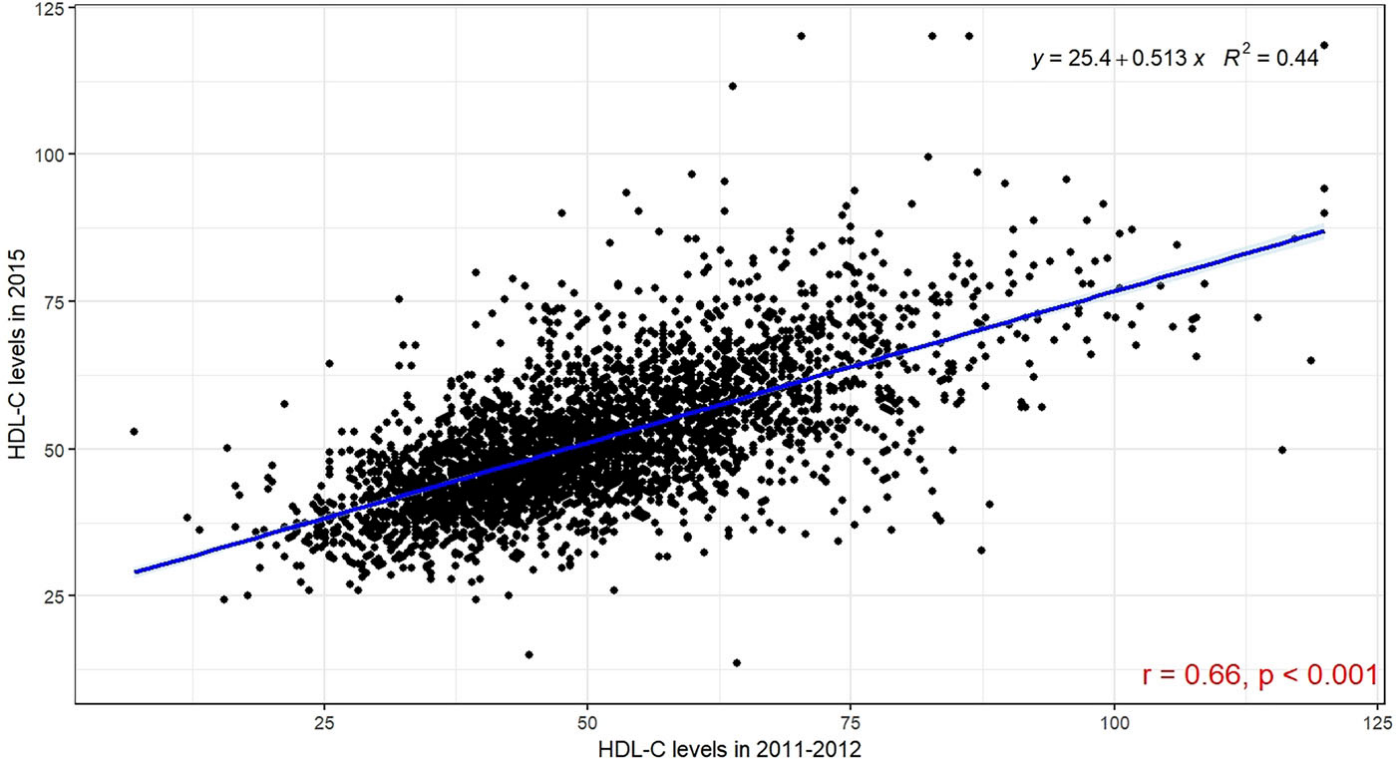
Scatter plot of the correlation between two HDL‐C measurements.

By employing time‐varying Cox regression models, we found that the impact of HDL‐C on the risk of stroke (HR 1.00, 95% CI 0.999–1.001 with *p* = 0.887) and all‐cause mortality (HR 1.00, 95% CI 0.9995–1.001 with *p* = 0.539) did not show a significant trend over time. At the same time, we also compared the HDL‐C levels in two blood tests conducted in 2011–2012 and 2015, and found a moderate positive correlation (*r* = 0.66, *p* < 0.05) in Figure 3.

### 3.4 Subgroup Analysis

**FIGURE 4:**
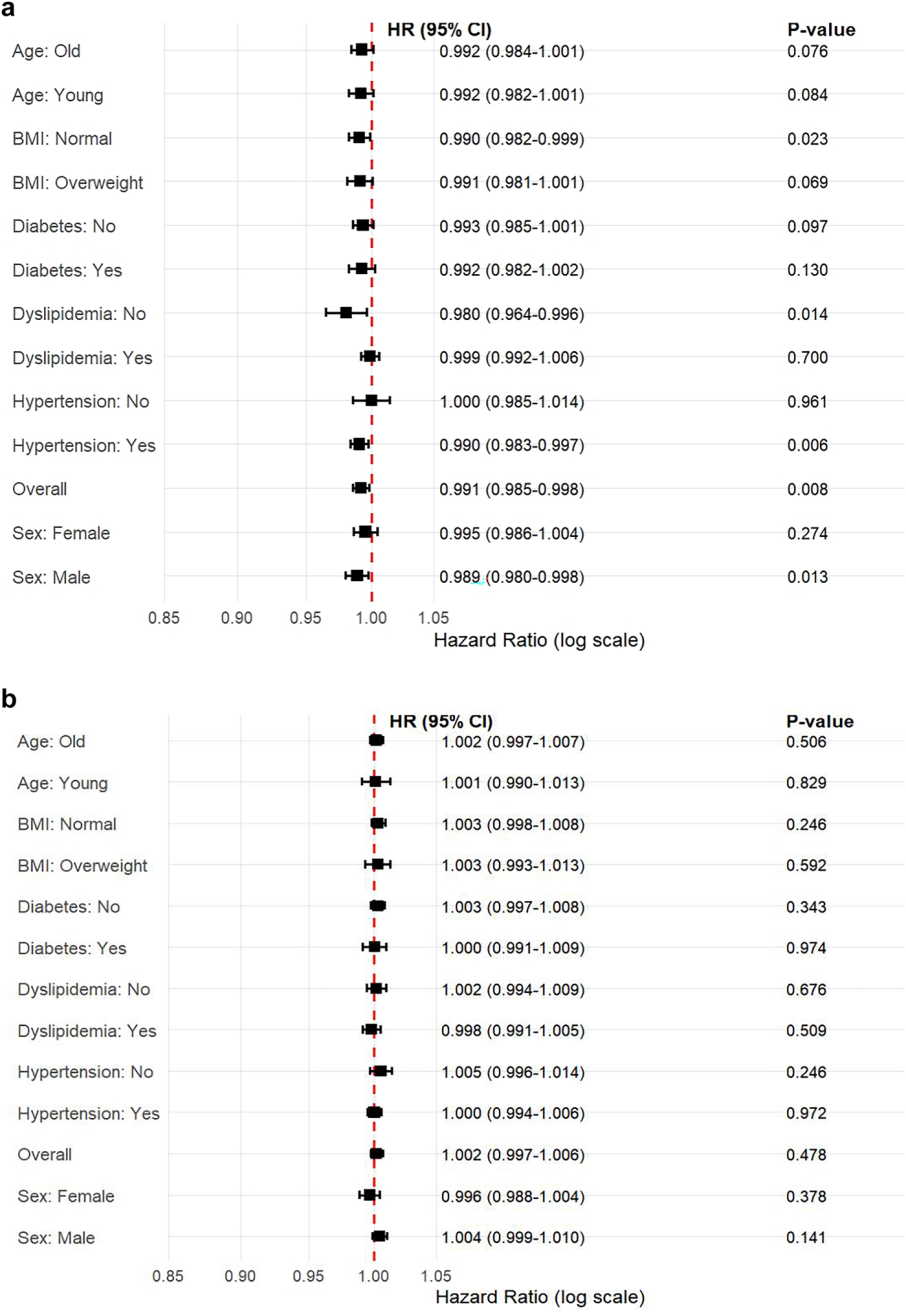
Subgroup analysis of the relationships between HDL‐C levels and stroke (A) and mortality (B).

In subgroup analysis, we defined age over 60 as old and BMI over 24 as overweight. For stroke occurrence, HDL‐C was a protective factor with statistical significance in the male group, the normal BMI group, the group without dyslipidemia, and the group with hypertension after adjusting for Model 3. There was no statistically significant association between HDL‐C levels and mortality risk in all predetermined subgroups of different ages, genders, BMI, and comorbidity status after adjusting in Model 3 (all *P* > 0.05). All results are shown in Figure 4.

### 3.5 Sensitivity Analysis

To evaluate the robustness of our findings, we performed sensitivity analyses using different covariate adjustments and excluding non‐stroke participants with baseline data only. Covariates were selected based on univariate/multivariate Cox analyses, and those with variance inflation factors > 5 were excluded to avoid multicollinearity. After adjusting for age, CRP, SBP, DBP, and lipid‐lowering therapy, HDL‐C remained a protective factor of stroke (HR 0.99, 95% CI 0.98–1.00, *P*  <  0.05). For all‑cause mortality, after adjusting for age, gender, smoking, and education status, Cys, CRP, SBP, BMI, Hb, and lipid‐lowering therapy, HDL‐C showed no significant association (HR 1.00, 95% CI 1.00–1.01, *p* = 0.83). Results were consistent after excluding non‐stroke populations with baseline participation only, confirming HDL‐C as a protective factor for stroke (HR 0.99, 95% CI (0.99–1.00), *P* < 0.05) but not for mortality (HR 1.00, 95% CI (1.00–1.01), *p* = 0.53) in Model 3.

### 3.6 Mediation Analysis

Mediation analysis revealed that BMI had a significant mediating effect between HDL‐C and stroke (total effect: −0.0027, average causal mediated effect: −0.0007, all *P* < 0.05), indicating that HDL‐C reduces stroke risk by lowering BMI. Approximately 25% of the protective effect of HDL‐C was achieved through BMI.

### 3.7 Competing‐Risk Model

In the Fine‐Gray model analysis, where stroke and death were treated as mutually competing events, HDL‐C remained a protective factor for stroke under the adjustment of Model 3, with an HR of 0.99 (0.99–1.00; *P* < 0.05). No statistically significant association was observed between HDL‐C and the risk of death (HR 1.00, 95% CI (1.00–1.01), *p* = 0.52).

## 4 Discussion

This study investigated the role of HDL‐C in stroke from both horizontal and vertical dimensions. It was found that HDL‐C was a protective factor against stroke among the middle‐aged and elderly population in China. BMI mediated the effect between HDL‐C and stroke, with a mediating proportion of 25%.

The aging population and the rise in metabolic diseases such as hypertension, DM, and dyslipidemia make stroke a huge disease challenge in China (Wang et al. 2022). Based on baseline data from 2011–2012, stroke patients were more likely to experience renal dysfunction and impaired glucose and lipid metabolism compared to non‐stroke populations. Follow‐up data indicated that patients who died had lower TG and LDL‐C levels and higher HDL‐C levels than those who survived. Considering the characteristics of this group, such as older age and lower BMI, their lipid metabolism may be affected by reduced nutrient intake, poor intestinal absorption, and slower metabolism.

Pharmacological studies have found no significant relationship between changes in HDL‐C levels caused by drug therapy and adverse cardiovascular outcomes, prompting researchers to re‐evaluate the role of HDL‐C (Lee et al. 2020; Riaz et al. 2019). Our study focused on the large database from China's middle‐aged and elderly population to re‐identify the relationships between HDL‐C and stroke and all‐cause mortality. We found a negative correlation between HDL‐C and overall stroke incidence, which was consistent with the results of a meta‐analysis including 18 studies. The study found that HDL‐C was negatively correlated with IS and subarachnoid hemorrhage, except for intracerebral hemorrhage (ICH). The negative correlation was related to HDL‐C's anti‐atherosclerosis and anti‐oxidative stress, while the positive correlation might be linked to its anti‐thrombosis, weakening platelet function, and promoting fibrinolysis, ultimately promoting the risk of ICH (Qie et al. 2021). Three studies of the National Health and Nutrition Examination Survey (NHANES) discovered increased levels of the dietary inflammatory index and decreased HDL‐C levels in both stroke and cardiovascular‐kidney‐metabolic syndrome (CKMS). Due to the close relationship between diet and blood lipids, this might suggest that diet‐related inflammation mediates the level and function of HDL‐C, further affecting the occurrence of stroke and CKMS (Zhao et al. 2025; Fan et al. 2025; Zhao et al. 2025). However, a study conducted in the NHANES found that HDL‐C levels below 1.55 mmol/L were negatively correlated with total stroke in the population over 40 years old, especially in men and Whites. Stroke risk increased with HDL‐C levels above 1.55 mmol/L, but this rise was not statistically significant (Hu et al. 2023). Data from Korea revealed that individuals with the highest increase in HDL‐C faced the greatest risk of CVD, suggesting the need to monitor changes in HDL‐C levels as a key indicator of disease risk (Kim et al. 2023). In addition, a prospective study from Tangshan, Hebei Province, China, uncovered a U‐shaped relationship between HDL‐C and stroke risk, affecting both ischemic and hemorrhagic types (Li et al. 2022). Therefore, researchers point out that the quantity of HDL‐C does not necessarily indicate the extent of its function. HDL‐C exhibits high heterogeneity in structure, composition, and function. As a primary subtype of HDL‐C, HDL2‐C demonstrates a negative correlation with carotid intima‐media thickness, which can promote the reverse transport of cholesterol, suggesting that it has a better anti‐atherosclerosis ability than HDL3‐C (Tiozzo et al. 2016). Additionally, HDL‐C is not static; its composition can change in response to fluctuations in the surrounding environment, which can affect its anti‐inflammatory, antioxidant, and cholesterol efflux abilities (Kontush et al. 2013; Holzer et al. 2015; Kostara et al. 2023; Stadler et al. 2021). HDL‐C gene mutations may also affect HDL‐C expression and dysfunction (Lin et al. 2024). Our research also found that BMI mediated the relationship between HDL‐C and stroke, and a study also found it in hypertensive individuals without atrial fibrillation (Yu et al. 2021). Obesity can affect the structure of HDL‐C, resulting in impaired HDL‐C function and weakened anti‐inflammatory and antioxidant functions (Stadler et al. 2021).

A cohort study involving 3.3 million people in China, a study from the UK Biobank database, a combined analysis of nine large cohort studies in Japan, and two prospective studies from the Copenhagen City Heart Study and the Copenhagen General Population Study all discovered a U‐shaped relationship between HDL‐C levels and all‐cause mortality, indicating that both low and ultra‐high HDL‐C levels were associated with an increased risk of death (Lu et al. 2023; Yuan et al. 2022; Hirata et al. 2018; Madsen et al. 2017). Low levels of HDL‐C could not guarantee its anti‐inflammatory, antioxidant, and anti‐atherosclerosis effects to play, while high levels of HDL‐C would bring about the remodeling of HDL‐C in structure and function, which meant that the normal function of HDL‐C was lost, and even turned into an “executioner” who promoted inflammation, cholesterol deposition, and atherosclerosis. As a result, researchers propose that future research should focus on understanding the different subtypes of HDL‐C and their specific functions (Razavi et al. 2024; Franczyk et al. 2021). However, in our study, the RCS plot did not reveal a statistically significant association between HDL‐C and all‐cause mortality after multivariable adjustment (overall P = 0.636), although a U‐shaped trend was visually suggested.

Our research has some limitations. Due to constraints in the database, we could not investigate the HDL‐C typing and function. Additionally, HDL‐C was measured at limited time points, which might cause regression‐dilution bias. Stroke outcomes were self‐reported rather than clinically validated, introducing differential misclassification bias. Residual confounders (such as unmeasured lifestyle factors like diet and exercise) might influence the results. Furthermore, the CHARLS database specifically targeted the middle‐aged and elderly population in China, and the age restriction (≥ 45 years) might limit applicability to younger populations.

## 5 Conclusions

In the Chinese population aged 45 and above, HDL‐C remains a protective factor for stroke, and BMI may mediate the relationship between HDL‐C and stroke. Attention should be paid to HDL‐C and BMI in the management of stroke.

## Author Contributions

**Zhi Liu**: conceptualization, data curation, formal analysis, methodology, writing – original draft. **Libo Liang**: conceptualization, data curation, formal analysis, investigation, writing – original draft. **Xiao Du**: project administration, supervision, writing – review and editing. **He He**: project administration, supervision, writing – review and editing, funding acquisition.

## Funding

This study was supported by the National Natural Science Foundation of China (Grant No. 32271494).

## Ethics Statement

All participants in the CHARLS study signed written informed consent, approved by the Biomedical Ethics Review Committee of Peking University (IRB00001052‐11015).

## Conflicts of Interest

The authors declare no conflicts of interest.

## Supporting information




**Supplementary Tables**: brb371179‐sup‐0001‐tables.docx

## Data Availability

The data that support the findings of this study are available from CHARLS's official website https://charls.pku.edu.cn/.
