# Supplementary material for: Association of HDL‐C Levels with Stroke and All‐cause Mortality: A Prospective Cohort Study from the CHARLS
Source: Brain Behav. 2026 Mar 10;16(3):e71179. doi: 10.1002/brb3.71179 (PMC12973163; doi:10.1002/brb3.71179)
Supplement: Supplementary file 1 — Supplementary Tables: brb371179‐sup‐0001‐tables.docx [file BRB3-16-e71179-s001.docx]

**Table S1.** Basic characteristics of the follow-up study from 2011 to 2020

|  | **Non-Stroke** | **Stroke** | **p** | **Alive** | **Death** | **p** |
| --- | --- | --- | --- | --- | --- | --- |
| N | 5562 | 528 |  | 5274 | 816 |  |
| BUN (mg/dL) | 15.24  [12.60, 18.29] | 15.45  [12.86, 18.12] | 0.352 | 15.18  [12.58, 18.12] | 15.78  [13.08, 19.12] | **<0.001** |
| UA (mg/dL) | 4.31  [3.57, 5.16] | 4.34  [3.56, 5.38] | 0.213 | 4.27  [3.55, 5.11] | 4.59  [3.77, 5.62] | **<0.001** |
| Cr (mg/dL) | 0.76  [0.66, 0.88] | 0.77  [0.67, 0.89] | **0.048** | 0.76  [0.64, 0.87] | 0.80  [0.69, 0.96] | **<0.001** |
| Cys (mg/L) | 0.98  [0.86, 1.13] | 1.01  [0.87, 1.17] | **0.003** | 0.97  [0.85, 1.10] | 1.14  [0.98, 1.35] | **<0.001** |
| TG (mg/dL) | 102.66  [73.46, 148.68] | 115.93  [85.85, 166.60] | **<0.001** | 104.43  [74.34, 151.34] | 101.78  [72.35, 143.37] | **0.015** |
| TC (mg/dL) | 190.98  [167.40, 216.11] | 194.27  [172.33, 220.85] | **0.012** | 191.37  [168.56, 216.50] | 190.59  [162.28, 218.53] | 0.144 |
| HDL-C  (mg/dL) | 50.26  [40.98, 60.70] | 46.78  [39.05, 55.67] | **<0.001** | 49.48  [40.59, 60.21] | 51.03  [41.37, 62.34] | **0.023** |
| LDL-C (mg/dL) | 115.59  [93.94, 138.02] | 118.88  [95.88, 141.50] | **0.023** | 116.37  [95.10, 138.79] | 113.66  [90.08, 139.27] | **0.019** |
| Glu (mg/dL) | 101.88  [94.14, 112.14] | 104.85  [96.66, 116.19] | **<0.001** | 102.06  [94.32, 112.14] | 102.78  [93.96, 116.14] | 0.098 |
| Hb (%) | 5.10  [4.90, 5.40] | 5.20  [4.90, 5.60] | **<0.001** | 5.10  [4.90, 5.40] | 5.20  [4.90, 5.50] | 0.364 |
| CRP (mg/dL) | 0.99  [0.54, 2.09] | 1.32  [0.68, 2.72] | **<0.001** | 0.98  [0.54, 1.97] | 1.41  [0.71, 3.68] | **<0.001** |
| eGFR (mL/min/1.73m²) | 93.63  [82.95, 101.68] | 91.12  [80.11, 98.97] | **<0.001** | 94.66  [84.32, 102.26] | 86.25  [72.25, 94.48] | **<0.001** |
| SBP (mmHg) | 127.33  [114.67, 142.67] | 135.00  [121.67, 151.33] | **<0.001** | 126.67  [114.67, 141.67] | 136.83  [122.00, 155.33] | **<0.001** |
| DBP (mmHg) | 74.33  [67.00, 82.67] | 78.00  [70.33, 87.67] | **<0.001** | 74.67  [67.00, 83.00] | 75.33  [67.00, 84.00] | 0.198 |
| Height (cm) | 157.50  [151.70, 163.90] | 157.95  [151.70, 164.30] | 0.354 | 157.50  [151.90, 164.00] | 157.50  [150.48, 164.00] | 0.135 |
| Weight (kg) | 57.20  [50.30, 65.10] | 60.30  [52.90, 69.08] | **<0.001** | 57.90  [51.10, 65.80] | 53.60  [46.98, 61.50] | **<0.001** |
| Waist (cm) | 84.20  [78.00, 91.60] | 88.75  [81.00, 96.20] | **<0.001** | 85.00  [78.00, 92.00] | 83.60  [76.80, 91.20] | **0.002** |
| BMI (kg/m^2^) | 22.97  [20.64, 25.53] | 24.04  [21.70, 26.95] | **<0.001** | 23.21  [20.94, 25.87] | 21.77  [19.70, 24.49] | **<0.001** |
| Age (years) | 59.00  [53.00, 68.00] | 61.00  [56.00, 69.00] | **<0.001** | 58.00  [52.00, 65.00] | 70.00  [63.00, 76.00] | **<0.001** |
| Smoking (%) |  |  | 0.051 |  |  | **<0.001** |
| Never | 3379 (60.8) | 299 ( 56.6) |  | 3304 (62.6) | 374 ( 45.8) |  |
| Quit | 485 ( 8.7) | 61 ( 11.6) |  | 419 ( 7.9) | 127 ( 15.6) |  |
| Current | 1698 (30.5) | 168 ( 31.8) |  | 1551 (29.4) | 315 ( 38.6) |  |
| Alcohol (%) | 1810 (32.5) | 168 ( 31.8) | 0.771 | 1696 (32.2) | 282 ( 34.6) | 0.186 |
| Female (%) | 2962 (53.3) | 272 ( 51.5) | 0.472 | 2924 (55.4) | 310 ( 38.0) | **<0.001** |
| Education (%) |  |  | 0.138 |  |  | **<0.001** |
| Primary or below | 3965 (71.3) | 388 ( 73.5) |  | 3668 (69.5) | 685 ( 83.9) |  |
| Middle or vocational | 1508 (27.1) | 137 ( 25.9) |  | 1521 (28.8) | 124 ( 15.2) |  |
| University or higher | 89 ( 1.6) | 3 ( 0.6) |  | 85 ( 1.6) | 7 ( 0.9) |  |
| Lipid-lowering therapy (%) | 865 (15.6) | 213 ( 40.3) | **<0.001** | 977 (18.5) | 101 ( 12.4) | **<0.001** |
| Hypertension (%) | 3343 (60.1) | 427 ( 80.9) | **<0.001** | 3200 (60.7) | 570 ( 69.9) | **<0.001** |
| DM (%) | 1427 (25.7) | 215 ( 40.7) | **<0.001** | 1420 (26.9) | 222 ( 27.2) | 0.900 |
| Dyslipidemia (%) | 2950 (53.0) | 396 ( 75.0) | **<0.001** | 2963 (56.2) | 383 ( 46.9) | **<0.001** |
| Liver disease (%) | 477 ( 8.6) | 90 ( 17.0) | **<0.001** | 502 ( 9.5) | 65 ( 8.0) | 0.175 |
| Kidney disease(%) | 754 (13.6) | 118 ( 22.3) | **<0.001** | 786 (14.9) | 86 ( 10.5) | **0.001** |
| Digestive disease(%) | 1918 (34.5) | 210 ( 39.8) | **0.017** | 1910 (36.2) | 218 ( 26.7) | **<0.001** |
| Heart problems(%) | 1347 (24.2) | 213 ( 40.3) | **<0.001** | 1359 (25.8) | 201 ( 24.6) | 0.517 |

BUN: blood urea nitrogen; UA: uric acid; Cr: creatinine; Cys: cystatin c; TG: triglycerides; TC: total cholesterol; HDL-C: high-density lipoprotein cholesterol; LDL-C: low-density lipoprotein cholesterol; Glu: glucose; Hb: glycosylated hemoglobin; CRP: C-reactive Protein; eGFR: estimated glomerular filtration rate; SBP: systolic blood pressure; DBP: diastolic blood pressure; BMI: body mass index; DM: Diabetes mellitus

**Table S2.** The HR (95% CI) of stroke according to HDL-C in three Models

| **Categories** | **Model 1** | **Model 2** | **Model 3** |
| --- | --- | --- | --- |
|  | **HR (95%CI) P value** | | |
| Continuous HDL-C per unit | 0.98 (0.98-0.99)  <0.001 | 0.99 (0.98-0.99) <0.001 | 0.99 (0.99-1.00) 0.008 |
| Quartile |  |  |  |
| Q1 | Ref. | Ref. | Ref. |
| Q2 | 0.94 (0.76-1.17)  0.60 | 0.99 (0.80-1.24)  0.96 | 1.09 (0.88-1.36) 0.45 |
| Q3 | 0.70 (0.55-0.88)  0.003 | 0.78 (0.61-0.99) 0.039 | 0.90 (0.71-1.15)  0.41 |
| Q4 | 0.55 (0.43-0.72)  <0.001 | 0.64 (0.49-0.83) <0.001 | 0.75 (0.58-0.99) 0.038 |

Model 1: unadjusted

Model 2: adjusted for age, gender, smoking, drinking, BMI

Model 3: Model 2 + adjusted for eGFR, CRP, lipid-lowering therapy

**Table S3.** The HR (95% CI) of mortality according to HDL-C in three Models

| **Categories** | **Model 1** | **Model 2** | **Model 3** |
| --- | --- | --- | --- |
|  | **HR (95%CI) P value** | | |
| Continuous HDL-C per unit | 1.01 (1.00-1.01) 0.017 | 1.00 (0.995-1.004)  0.92 | 1.00 (1.00-1.01) 0.482 |
| Quartile |  |  |  |
| Q1 | Ref. | Ref. | Ref. |
| Q2 | 0.94 (0.77-1.15)  0.53 | 0.98 (0.80-1.20)  0.84 | 1.01 (0.83-1.24)  0.90 |
| Q3 | 1.04 (0.85-1.26)  0.70 | 0.94 (0.77-1.15)  0.54 | 1.01 (0.82-1.23)  0.96 |
| Q4 | 1.22 (1.01-1.47) 0.037 | 1.00 (0.82-1.22) 0.999 | 1.09 (0.89-1.33)  0.40 |

Model 1: unadjusted

Model 2: adjusted for age, gender, smoking, drinking, BMI

Model 3: Model 2 + adjusted for eGFR, CRP, lipid-lowering therapy
